# Rapid and Effective Recovery of Oleanolic and Maslinic Acids from Olive Leaves Using SFE and pH-Zone Centrifugal Partition Chromatography

**DOI:** 10.3390/molecules30132709

**Published:** 2025-06-24

**Authors:** Lemonia Antoniadi, Apostolis Angelis, Theodora Nikou, Dimitris Michailidis, Leandros A. Skaltsounis

**Affiliations:** 1 PharmaGnose S.A., 57th km Athens-Lamia National Road, 32011 Oinofyta, Greece; monikaant@pharm.uoa.gr (L.A.); michailidis@pharmagnose.com (D.M.); 2 Division of Pharmacognosy and Natural Products Chemistry, Department of Pharmacy, National and Kapodistrian University of Athens, Panepistimioupoli Zografou, 15771 Athens, Greece

**Keywords:** oleanolic acid, maslinic acid, SFE, co-solvent, pH-zone refining CPC, enzymatic activity

## Abstract

Olive leaves, the main byproducts of olive cultivation, are characterized by a plethora of bioactive metabolites with significant nutritional value. Their main pentacyclic triterpenes, Oleanolic Acid (OA) and Maslinic Acid (MA), are two high added-value compounds with remarkable activities. This study aimed to develop an efficient methodology for extracting and purifying OA and MA, utilizing Supercritical Fluid Extraction (SFE) and Centrifugal Partition Chromatography (CPC)—two modern, scalable, and green techniques. A total of 21 g of olive leaves were subjected to SFE using supercritical CO_2_ and ethanol as co-solvent. The extraction employed a step gradient mode, starting with 100% CO_2_ and incrementally increasing ethanol (0–10% *w*/*w*) every 20 min. Fractions rich in OA and MA (500 mg) were further purified via CPC, utilizing pH zone refining to exploit the protonation and deprotonation properties of acidic triterpenes. The biphasic solvent system consisted of *n*-hexane, ethyl acetate, ethanol, and water (8:2:5:5 *v*/*v*/*v*/*v*), with trifluoroacetic acid added to the stationary phase and triethylamine added to the mobile phase. This two-step process yielded 89.5 mg of OA and 28.5 mg of MA with over 95% purity, as confirmed by HPLC-ELSD and ^1^H-NMR. Moreover, purified compounds and SFE fractions exhibited promising elastase and collagenase inhibition, highlighting them as dermocosmetic agents.

## 1. Introduction

Olive tree (*Olea europaea* L.) cultivation is considered the most important agricultural activity of the Mediterranean region. Olive leaves are the main byproducts of olive harvesting and comprise almost 10% of the materials gathered from olive mills. A plethora of bioactive secondary metabolites found in olive leaves, which provide health promoting benefits [1], attract the scientific community and the desire of the industry to valorize these byproducts. The abundance of valuable phenolic compounds has directed the majority of scientific research to focus on them. Consequently, research on olive leaves has highlighted that a chemical group, namely biophenols, is closely linked with the structural diversity and the huge broad variety of biological activities [2]. Despite the considerable nutritional and biological significance of olive biophenols, triterpenoids—an important chemical group—are often overlooked. The epidermis of olive leaves consists of sterols and this type of terpenoids, like triterpenic acids, in high levels [3,4].

Oleanolic acid (OA) and Maslinic acid (MA) are the main and most representative pentacyclic triterpenic b-amyrin derivatives. These acids biosynthetically derive from the cyclization of squalene. During the olive’s developing stage, sterols (primary metabolites) and non-steroidal triterpenoids (secondary metabolites) share oxidosqualene as a common precursor. The enzyme β-amyrin synthase catalyzes its cyclization into β-amyrin, and further oxidation gives rise to the triterpenic dialcohol erythrodiol, generating the hydroxyl pentacyclic triterpenic acids, OA and MA [5,6]. The presence of a hydroxyl group at position 2 of the pentacyclic skeleton is the only difference in the chemical structure between these acids.

Oleanolic acid (3β-hydroxy-olean-12-en-28-oic acid) has been associated with anti-inflammatory activity due to the inhibition of COX-2, which decreases the production of prostaglandin PGE2 [7]. It seems that the additional inhibitory effect against CYP1A2 may connect to its anti-inflammatory and anticancer effects [8]. The gastroprotective and antiulcerogenic properties of various compounds have been extensively studied over several decades. These effects are closely linked to the previously described well-characterized pharmacological properties [9]. It stimulates the release of NO and TNF-a, including induction of iNOS activity and TNFa expression in macrophages [10]. OA has also been investigated for the hepatoprotective effect with promising results. [11,12]. Additionally, there is evidence related to antiviral activity, including anti-HIV activity by inhibition of HIV-1 protease [13].

Maslinic acid [(2α,3β)-2,3-dihydroxyolean-12-en-28-oic acid] is an established highly antioxidant agent. Its main properties are the decrease of the susceptibility of plasma and hepatocyte membranes to lipid peroxidation [14]. Also, MA could become involved in oxidative stress modulating the release of oxygen reactive species, such as NO and H_2_O_2_ [15]. The anti-inflammatory effects of MA could be interceded by NF-B pathway inhibition and effectively inhibit the production of the proinflammatory cytokines TNF- and ILs in LPS stimulated macrophages [16]. MA has further been reported to suppress nuclear factor-kappa B (NF-B) regulated osteoclastogenesis in bone marrow monocytes and to inhibit TNF-induced NF-B [17]. The antitumor activity of MA has become remarkable in recent years, and the majority of research articles correspond to in vitro and in vivo experiments that show the anti-proliferative and/or pro-apoptotic effect, with different involved signaling pathways [18,19]. The role of MA in glucose metabolism has also been extensively studied and has demonstrated an acclaimed pharmacological inhibitor of glycogen phosphorylase (GP) [20], which showed an auspicious antidiabetic potential [21]. Historically, MA has been considered a potent antiparasitic compound, and many semisynthetic compounds derived from MA have also demonstrated anti-HIV activity by inhibiting the human immunodeficiency virus (HIV-1) protease [22]. Moreover, concerning dermocosmetic interest, several studies demonstrated that MA and OA as well are potential nutraceutical agents with activity against many enzymes such as tyrosinase, elastase, and collagenase [23].

In accordance with the bibliography, there is significant research focusing on the development of procedures for isolating OA and MA from olive products. A resent research study optimized flash chromatography for the separation of OA and MA from olive leaf extracts. Using *n*-hexane and ethyl acetate as mobile phases, the method achieved 109 mg of pure OA from 10 g of plant material (10.9 mg/g) and 6.25 mg/g of pure MA, confirmed via ATR-FTIR and NMR spectroscopy. This approach offers a rapid and efficient alternative to traditional extraction methods [24]. Huang et al. developed a continuous HSCCC method with an elution–extrusion mode to separate MA and OA from olive pulp extracts. The process achieved 271.6 mg of MA with 86.7% purity and 83.9 mg of OA with 83.4% purity. This method allows for large sample loading and efficient separation within 120 min [25]. A study explored the use of aqueous solutions of surface-active ionic liquids for extracting OA from dried olive leaves. Imidazolium-based ionic liquids with long alkyl chains enhanced the solubility of OA in water, achieving extraction yields of up to 2.5 wt.%. This method presents an eco-friendly alternative to traditional organic solvents [26]. A comparative study evaluated various extraction methods for triterpenic acids from olive fruits. Ultrasound-assisted extraction using ethanol or ethanol: methanol mixtures resulted in significantly higher yields of OA and MA compared to other methods. Additionally, freezing or lyophilizing the olive material prior to extraction preserved higher triterpenic acid content [27]. In addition, a patent has been also reported for the preparative isolation of the two acids from the milling waste products of olive, obtained in high yields and purity [28]. Another patent describes a method for preparing OA and MA involving solvent extraction with mixtures such as chloroform and methanol, followed by purification steps like column chromatography and recrystallization to obtain high-purity compounds [29].

Supercritical Fluid Extraction (SFE) is a versatile and green technique for the extraction of natural products and is widely recognized for its efficiency, sustainability, and adaptability across a range of applications [30]. The method uses Supercritical Fluids (SCFs), which exhibit unique physicochemical properties that combine the density and solvating power of a liquid with the diffusivity and viscosity of a gas. These properties enable effective penetration into plant matrices and rapid dissolution of bioactive components, leading to enhanced recovery of target compounds under mild operating conditions.

Carbon dioxide (CO_2_) is the most commonly used SCE solvent due to its numerous advantages: it is non-toxic, non-flammable, chemically inert, inexpensive, and readily available. Importantly, CO_2_ leaves no solvent residues in the final extract, ensuring product safety and purity. Its relatively low critical temperature (31.1 °C) and pressure (73.8 bar) make it ideal for processing thermolabile and sensitive bioactive components, such as polyphenols, terpenoids, and essential oils.

Another major benefit of SFE is the tunability of solvent properties by manipulating pressure and temperature or by adding co-solvents such as ethanol. This flexibility allows selective extraction of compounds based on polarity, enabling the isolation of specific phytochemical groups. For example, the incorporation of ethanol can enhance extraction of polar secondary metabolites, improving both yield and composition. SFE has successfully been applied to obtain enriched extracts of oleuropein from olive leaves [31] and has also been used to target diverse compounds including aliphatic hydrocarbons [32], squalene [33], tocopherols [33,34], mannitol [35], and various phenolics [36]. Additionally, the technique has gained attention for the extraction and purification of triterpenic acids from various plant matrices [37,38], offering a clean, efficient alternative to conventional solvent-based methods.

Centrifugal Partition Chromatography (CPC) is a solid support-free liquid–liquid separation technique [39] that operates on the principle of partitioning compounds between two immiscible liquid phases based on their partition coefficients. CPC uses a hydrostatic column composed of a cascade of oval twin-cells, enabling large sample capacity and scalable preparative purification of secondary metabolites [39,40]. A notable variation, the pH-zone refining mode of CCC, enhances separation of ionizable compounds such as organic acids, alkaloids, and triterpenoids [41,42,43,44,45]. This mode uses a pH gradient created by adding a retainer (e.g., TFA or TEA) in the stationary phase and an eluter (e.g., NH_3_, Na_2_CO_3_, or HCl) in the mobile phase. With proper tuning of the retainer/eluter ratio, the technique provides sharp separation boundaries and high-resolution isolation, even at preparative scale [46,47,48]. While pH-zone refining CPC has been applied to the isolation of Oleanolic acid (OA) [49], its application to Maslinic acid (MA) or mixed extracts containing both triterpenoids remains unexplored. Thus, the isolation and purification of these pentacyclic acids from olive leaves is regarded as a highly challenging task, due to their complex structure and biological significance as well. Taken also into consideration are the industry needs for compounds in high quantities and purities employing eco-friendly procedures and the development of a rapid versatile and “green” method in preparative scale, incorporating the use of special methodologies like SFE and CPC. In the current approach, the ability of triterpenoid acids to protonate or deprotonate under pH variations was taken as an advantage to develop a pH zone refining mode and would be exaggerated enough to combine this technique of CPC with SFE extraction in order to separate OA and MA from olive leaves. Moreover, MA, OA, and other SFE fractions were tested against significant dermocosmetic enzymes, such as elastase and collagenase, revealing a possible very promising application for cosmetic industry.

## 2. Results

### 2.1. Recovery of Enriched Fraction in OA and MA Using SFE

**Figure 1:**
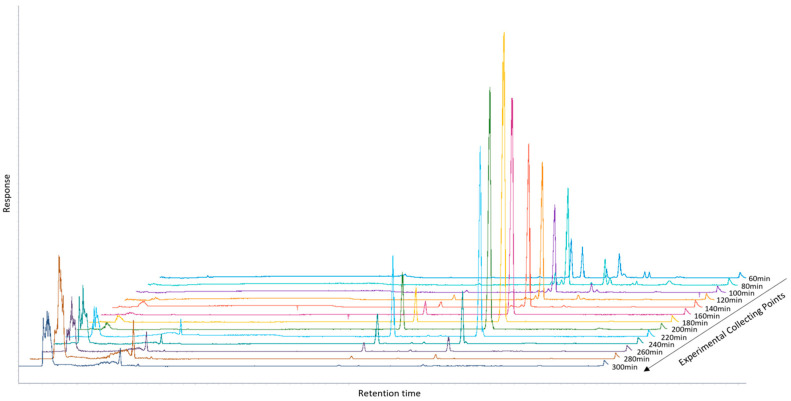
HPLC-ELSD chromatograph of leaves’ extracts in all collected time points.

Olive products, like leaves and olive oil, are very complex mixtures of many different chemical categories of secondary metabolites [43] with a broad range of skeletons and polarities. Thus, their targeted fractionation is a challenging procedure in order to achieve a successful separation of the main metabolites. SFE has been applied to olive leaves, yielding significant insights into the efficiency of this technique for fractionation and the recovery of triterpenic acids, specifically OA and MA [38]. Ethanol was employed as a co-solvent in the SFE procedure, with its concentration gradually increasing from 0% to 10% with a flow rate of CO_2_ approximately at 0.24 kg/h. This stepwise adjustment was implemented to systematically enhance the polarity of the supercritical CO_2_, allowing for the selective extraction of compounds across a broad range of polarities. At low ethanol concentrations (e.g., 2.5%) and with no ethanol use (e.g., 0%), non-polar and slightly polar compounds were primarily extracted, while the progressive increase to higher concentrations (5% and 7.5%) facilitated the recovery of more polar metabolites, including OA and MA. At each CO_2_-to-EtOH ratio, samples were collected in 20 min intervals. Consequently, three fractions were obtained from each ratio. The most promising fractions, based on their composition and bioactive content, were observed at ethanol concentrations of 5% and 7.5%, as demonstrated in Figure 1 through chromatographic analysis.

The fractions collected at these concentrations showed a higher enrichment of triterpenic acids, such as OA and MA, indicating an optimal balance between solvent polarity and compound solubility. These findings suggest that the ethanol percentage significantly influences the selectivity and efficiency of the extraction process. It is worth noting that both OA and MA exhibit remarkable thermal stability, as supported by literature data indicating no significant degradation below 50–60 °C [50,51]. Therefore, the temperature of 35 °C applied during the Supercritical Fluid Extraction (SFE) process in our study falls well within the established safe range for preserving the structural integrity of these triterpenoids. Moreover, the absence of detectable degradation products in the HPLC chromatograms further confirms the thermal stability of OA and MA under the conditions employed.

The extraction yields of the collected fractions are summarized in Table 1, highlighting the superior recovery rates achieved at 5% and 7.5% ethanol. This data underscores the importance of optimizing co-solvent concentration to maximize the isolation of target bioactive compounds, while minimizing undesired compounds. By systematically varying the ethanol ratio and employing time-controlled fractionation, the process provided valuable insights into the relationship between extraction parameters and the quality of the recovered fractions.

### 2.2. Purification of Triterpenic Acids Using Refining pH-Zone CPC

The ability of triterpenoid acids to protonate or deprotonate under pH variations was taken as an advantage for the selection of the appropriate biphasic system. Thus, the choice of the system for the analysis of the selected fractions enriched in OA and MA was based on a recently described method used for the pH-Zone CPC fractionation of cannabinoid acids [52]. To evaluate the suitability of the selected solvent systems and the overall methodology, HPTLC analysis was performed under both neutral and alkalinized biphasic conditions. The analysis revealed a distinct shift of the targeted triterpenic acids from the upper to the lower phase upon alkalinization (Figure S2, Supplementary Materials), indicating successful partitioning behavior under the modified conditions. These results demonstrate the effectiveness of the alkalinized biphasic system in selectively isolating the target compounds and validate the method as efficient for the purification of both triterpenic acids from the enriched SFE fractions. It is worth noting that both OA and MA are known to be chemically stable under mildly acidic to basic conditions within the specified concentration range. Accordingly, no degradation products were detected in the chromatographic profiles following CPC separation, indicating that the structural integrity of the triterpenic acids was maintained throughout the process.

The pH-Zone refining mode CPC analysis was run in a 200 mL CPC column in descending mode. After filling the column with the acidified upper phase with 100 mM of TFA (stationary phase), a 500 mg sample was injected, and the alkalified lower phase with 80 mM of TEA (mobile phase) was pumped through the column. The whole procedure was monitored by TLC analysis and all fractions containing the pure targeted compounds were evaporated to dryness.

The fractions containing the isolated MA (fractions 24–25) were evaporated to dryness giving finally 28.5 mg. Similarly, 89.5 mg of pure OA was isolated from fractions 35–36. The high purity of the recovered compounds was confirmed by HPLC-ELSD analysis (Figures S3 and S5, Supplementary Materials). The structural elucidation of them was carried out using experimental ^1^H-NMR spectra (Figures S4 and S6, Supplementary Materials), and the obtained spectra were compared with literature data for confirmation [53]. It is worth noting that although their yields obtained with this current methodology were lower than those reported by applying other methods such as flash chromatography, the trade-off is justified by the significantly higher purity, reduced solvent consumption, and environmentally friendly approach of SFE-CPC, aligning with green chemistry principles and offering a more sustainable alternative for industrial applications [24]. To assess the overall efficiency of the extraction and purification process, the content of OA and MA in the raw olive leaf material was quantified using HPLC analysis based on external calibration curves. The dried leaves contained 10.08 mg/g of OA and 4.42 mg/g of MA, corresponding to a total of 211.68 mg OA and 92.82 mg MA in the 21 g of starting material. Based on the isolated amounts (89.5 mg OA and 28.5 mg MA), the recovery rates were calculated to be approximately 42.3% for OA and 30.7% for MA. These values confirm that the method is effective for selectively enriching these triterpenic acids, while maintaining high purity and adhering to green processing principles (Figure S7, Supplementary Materials).

### 2.3. Biological Evaluation Against Elastase and Collagenase Enzymes

Elastase is a member of the serine protease family, responsible for tissue dissociation. Its inhibition may influence various biological processes, including wound healing and the reduction of skin wrinkles [54,55,56]. Similarly, collagenase, a metalloproteinase, plays a critical role in the breakdown of collagen within the extracellular matrix. Factors such as sun exposure and aging significantly contribute to the overexpression of these enzymes, disrupting the system’s balance and ultimately accelerating skin aging [57,58,59].

The enriched fraction obtained from SFE, along with the pure compounds OA and MA, were evaluated for their inhibitory effects on elastase and collagenase. The IC_50_ value of a positive control was included in both experiments to ensure the reliability of the procedures. All samples were tested at three different concentrations in triplicate.

**Figure 2:**
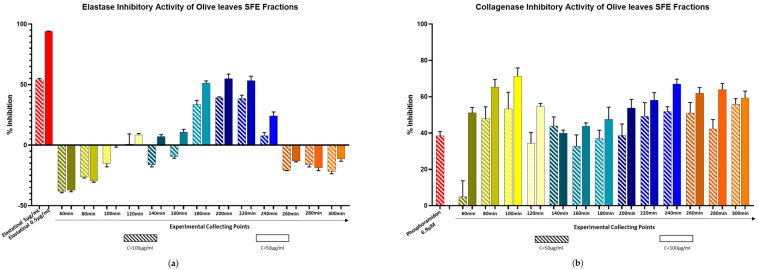
(**a**) Elastase inhibitory activity of Olive leaves SFE fractions and (**b**) Collagenase inhibitory activity of Olive leaves’ SFE fractions. Bars represent mean ± S.D., *n* = 3.

Interesting results emerged from the elastase inhibition experiments conducted on SFE fractions. As shown in Figure 2a, the first and last fractions of the extraction process exhibited no detectable elastase inhibitory effects across the three tested concentrations. However, fractions collected during the middle stages of the extraction, specifically at 180, 200, 220, and 240 min, demonstrated remarkable inhibitory activity. This observation correlates closely with the CO_2_-to-EtOH ratio employed during these specific time intervals. The fractions displaying significant elastase inhibition corresponded to the extraction phases, where ethanol concentrations were adjusted to 5% and 7.5%, enhancing the polarity of the supercritical CO_2_. Notably, these fractions also contain the triterpenic acids of interest. These results are in agreement with literature data as OA and MA have already been reported as potent elastase inhibitors [60,61]. To confirm these data, the parameters were repeated by testing the pure substances, from which the bibliographic data were confirmed. (Figure S8, Supplementary Materials). The tested IC_50_ of OA was calculated at 17 μM.

The results of the collagenase activity assay were noteworthy, due to the fact that all tested concentrations of SFE fractions showed an incredible inhibition. (Figure 2b). In addition to that, pure compounds were tested, and it was proved that both are inhibitors. (Figure S9, Supplementary Materials). It must be emphasized that these experiments were performed for the first time against collagenase enzyme, especially at the substrate MMP2. Previous evaluations referred to another substrate MMP1. These substrates belong to the metalloproteinases, but they have severe differences in their structure and three-dimensional structure [54]. Although the current study focused on the in vitro enzymatic inhibition activity of Oleanolic and Maslinic acids, future investigations will be directed toward validating these effects in cell-based models, such as human dermal fibroblasts. Such studies will provide further insight into the compounds’ anti-aging potential and support their application in dermocosmetic formulations.

## 3. Materials and Methods

### 3.1. Materials and Reagents

The olive leaves were collected in October 2024 and were provided by Polychroniadi Niki. After air-drying, leaves were ground in a mill until a fine powder was obtained, and finally, were stored at room temperature.

For SFE and CPC experiments, analytical grade solvents *n*-Hexane (*n*-Hex), Ethyl Acetate (EtOAc), and Ethanol (EtOH) were purchased from Carlo Erba Reactifs SDS (Val de Reuil, France), while Trifluoroacetic acid (TFA) and Triethylamine (TEA) were obtained from Sigma-Aldrich (Steinheim, Germany). The aqueous solutions were prepared by using deionized water. For HPTLC analysis, Methanol (MeOH), Dichloromethane (DCM), and Toluene were obtained from Fisher Scientific (Pittsburgh, PA, USA), while Sulphuric Acid (H_2_SO_4_) and Vanillin standard (98%) were purchased from Sigma-Aldrich (Steinheim, Germany). For HPLC analysis, Water, MeOH, acetonitrile and acetic acid were obtained from Fisher Scientific (USA). In detail, for enzymatic assays, elastase type IV from porcine pancreas (EC Number 254-453-6), *N*-Succinyl-Ala-Ala-Ala-p nitroanilide (EC Number 257-823-5), Trizma base reagent grade, elastatinal, collagenase from Clostridium histolyticum (released from physiologically active rat pancreatic islets Type V, ≥1 FALGPA units/mg solid, >125 CDU/mg solid, EC Number: 232-582-9), MMP 2 substrate fluorogenic, and phosphoramidon were purchased from Sigma-Aldrich. H_2_O was obtained from the Milli-Q purification system (Merck Millipore, Darmstadt, Germany).

### 3.2. Extraction of Olive Leaves Using Laboratory SFE

The Supercritical Fluid Extraction was performed on an SFE 1–2, SEPAREX lab scale apparatus (SEPAREX, Champigneulles, France) designed to allow the study of a wide range of conditions. It consisted of a CO_2_ tank, a liquid CO_2_ pump (that can deliver up to 10 kg/h), an extraction vessel (100 mL), a separator (with 200 mL capacity), a co-solvent pump (with 10 mL/min maximum flow rate), and a cooling system using glycol.

A precursory experiment was carried out using this lab-scale SFE apparatus, with a view to evaluate the effect of ethanol as a co-solvent at different proportions (i.e., 0%, 2.5%, 5%, 7.5%, and 10% *w*/*w*). The experiment was performed by filling the extraction vessel (100 mL) with 21 g of dried olive leaf material. The pressure in the extraction vessel was kept constant at 15 MPa, while the pressure in the separator’s compartment was held at 4 MPa. The extraction and separation temperatures were set at 35 °C and 30 °C, respectively. This temperature was chosen not to risk the stability of thermolabile components. The extract was partially collected 60 min after the beginning of the procedure and then every 20 min for each proportion of EtOH addition. The whole SFE procedure lasted approximately 5 h. Finally, all the received extracts were evaporated, and the total dried weight was calculated.

### 3.3. Fractionation of Triterpenic Acids Using Refining pH-Zone CPC

The fractionation procedure was performed on the laboratory scale FCPC200 instrument from Rousselet-Robatel Kromaton (Annonay, France), equipped with a 200 mL column (Angers, France). The experiments were conducted at a rotation speed from 500 to 1000 rotation per minute (rpm); thus, the relative centrifugal acceleration in the partition cells reached up to 437 g. The two immiscible phases were expelled from a Prep 36 Lab Alliance dual piston pump (State College, PA, USA). The enriched fraction was injected into the column through a 10 mL loop, and the fractions were collected by a Büchi B-684 fraction collector (Flawil, Switzerland). All experiments were carried out at room temperature (20 ± 2 °C).

The selected solvent system (1 L) was prepared by mixing *n*-Hex, EtOAc, EtOH, and Water in a ratio of 8:2:5:5 (*v*/*v*/*v*/*v*). The two phases of each system were separated after equilibration of the mixture. Then, 8 mL of TFA were added to the organic phase to achieve a final concentration of 100 mM with a pH value of 2, while 11 mL of TEA were added to the aqueous phase to adjust the pH to 10, resulting in a TEA concentration of 80 mM in the final solution. The experiment was performed in descending mode, so the nonpolar phase was the static phase and the polar phase was the mobile phase, respectively. Initially, the column (200 mL FCPC200^®^, Rousselet Robatel, Annonay, France) was fulfilled with the acidified static phase, the speed of rotation was adjusted and maintained constant throughout the experiment at 750 rpm, and the flow rate was maintained at 5 mL/min. Then, 250 mg of combined SFE fractions (enriched in OA and MA) (dissolved in 10 mL of a 60:40 *v*/*v* mixture of neutral mobile (lower) phase: acidic static (upper) phase) were injected into the column via a 10 mL loop and eluted with the alkalified mobile phase at the same initial conditions. The fraction collector was set to collect 10 mL fractions during the experiment. After initiation, 40 fractions (elution step) were collected, and the content of the column was extruded using the organic neutral phase of the biphasic system in descending mode. An additional 20 fractions were collected during the extrusion phase. The experiment lasted 100 min, while all collected fractions (60 fractions) were analyzed by TLC and HPLC-ELSD and pooled, based on their chemical similarity, resulting in 13 combinations.

### 3.4. HPLC-DAD-ELSD, HPTLC and NMR Profiling

The HPLC-DAD-ELSD analysis of SFE enriched fractions and fractions from CPC was carried out on an Agilent 1260 Infinity II system equipped with a 1260 Infinity Degasser, a 1260 Infinity II Quaternary pump, a 1260 Infinity II Vial sampler with thermostat, a 1260 Infinity II Multicolumn Thermostat, a 1269 Infinity II Diode Array Detector, and a 1260 Infinity II Evaporative Light Scattering Detector (Agilent, Santa Clara, CA, USA). Agilent OpenLab CDS software (version 2.8) was used for the operation of the system and data processing. Samples were injected into a reversed-phase Phenomenex Hypersil 5 um C18-BDS column (Torrance, CA, USA, 250 × 4.00 mm, 5 micron), and the compounds were eluted using a gradient mode of 1% acetic acid in water: MeOH in a ratio 85:25 (*v*/*v*) (solvent A) and Acetonitrile (solvent B). Separation was achieved under the following gradient conditions: 10% to 50% B at the first 15 min, followed by a linear gradient to 100% B until 40 min, a return to the initial conditions, and then, isocratic conditioning at 10% B for 5 min. The total run time was 50 min, and UV spectra were recorded from 200 to 400 nm, while the PDA was set at 210, 256, and 280 nm for all samples. The column temperature was kept stable at 20 °C. The flow rate was 0.80 mL/min, and the injection volume was 25 μL. Also, analytes were monitored by ELSD. The evaporator temperature was 50 °C, and the nebulized temperature was 80 °C, while the nitrogen flow rate was set at 2 mL/min.

For the HPTLC analysis of the enriched fractions, a concentration 10 mg/mL in EtOAc was prepared, and 25 μL of each sample were applied onto 20 × 10 cm HPTLC plates (silica gel 60 F254, 0.20 mm layer thickness; Merck, Darmstadt, Germany) using the Automatic TLC sampler (ATS4, CAMAG, Muttenz, Switzerland). The software platform used was VisionCats 2.5 (CAMAG) with the following specific settings: tracks with 7.0 mm bands, 8 mm distance from the lower edge, 11.4 mm from the left and right edges, and 9.4 mm between the different tracks. The application of samples was carried out using a 25 μL syringe (Hamilton, NV, USA), and the air was provided by an aspirator. The plates were developed with an automatic chamber (ADC2, CAMAG) using standard settings: 20 min chamber saturation with filter paper, 10 min of plate conditioning at 33% relative humidity using MgCl_2_ as a desiccant, and 5 min of plate drying. The first development system consisted of DCM/MeOH (95:5 *v*/*v*), and the second was Toluene/MeOH (95:5 *v*/*v*). Plate images at 254 nm and 366 nm were performed on a Visualizer 2 Documentation System (CAMAG). For visualization of the spots, the HPTLC plates were sprayed with the derivatization reagent, vanillin reagent, which was prepared by dissolving 1 g of vanillin in 100 mL of EtOH 96% and carefully adding 2 mL of H_2_SO_4_.

NMR spectra were recorded at 300 K on a Bruker Avance III 600 spectrometer (Bruker Biospin GmbH, Rheinstetten, Germany), operating at 600.11 MHz and equipped with a 5 mm PABBI 1 H/D-BB inverse detection probe with a z-gradient. All 1D (1H) experiments were carried out by using default Bruker pulse experiments with standard acquisition parameters, while the solvent used was chloroform (99.8 atom% D; Euriso-Top, Saclay, France). The spectra were referenced to the residual solvent signals at 7.26 and 77.0 ppm for ^1^H and ^13^C, respectively. Data acquisition and processing were performed using TopSpin 2.1 software (Bruker Biospin GmbH).

### 3.5. Enzymatic Assays

#### 3.5.1. Elastase Assay

Elastase inhibitory activity was measured by the presence of p-nitroaniline, after the reaction of the elastase enzyme with the substrate *N*-succinyl-Ala-Ala-Ala-p-nitroanilide, using a previously described protocol with the minimum of modifications [62,63]. Initially, 70 μL of Trizma-base buffer (50 mM, pH 7.4), 10 μL of the tested sample, and 5 μL of elastase solution (0.50 U/mL, dissolved in Trizma-base buffer containing < 5% organic solvent) were mixed in a 96-well microplate and incubated for 10 min at 25 °C, avoiding light exposure. Following this procedure, 15 μL of the substrate (2 mM dissolved in Trizma-base buffer) were added in each well, and then, the plate was incubated for 30 min at 37 °C. The presence of p-nitroanilide was measured spectrophotometrically at 405 nm by Tecan (Infinite 200 Pro series, Männedorf, Switzerland). The final tested concentrations of the extracts were 50 μg/mL, 100 μg/mL, and 300 μg/mL dissolved in Trizma-base buffer, the concentrations of the pure compounds were 5 μM, 10 μM, 25 μM, 50 μM, 100 μM, and 200 μM, and the percentage of DMSO was under 5% of the total volume inside the plate. The negative control of the assay was 5% DMSO Trizma-base buffer, while the positive was Elastatinal (IC_100_ = 5 μg/mL and IC_50_ = 0.5 μg/mL), a strong irreversible competitive elastase inhibitor. Experiments were performed in triplicate.

The elastase inhibitory activity was calculated by the following equation:

Inhibition (%) = (Abs_A_ − Abs_B_)/(Abs_A_) × 100 (1)

where Abs_A_ = Abs_Control_ − Abs_Control Blank_ and Abs_B_ = Abs_Sample_ − Abs_Sample Blank_. Abs_Control_ is the absorbance of Trizma-base Buffer with 5% DMSO, elastase enzyme, and substrate, and Abs_Sample_ is the absorbance of the tested sample in the Trizma-base buffer, elastase enzyme, and substrate. Blanks consist of all the above reagents except the enzyme.

#### 3.5.2. Collagenase Assay

Collagenase inhibitory activity was determined by a previously described enzymatic method with slight modifications, based on the fluorescence of the MMP-2 substrate after its degradation by collagenase [62]. In a 96-well black microplate, 50 μL Tris-HCL buffer (10 mM, pH = 7.3), 25 μL of tested sample (dissolved in Tris-HCL buffer), and 25 μL of collagenase solution (40 μg/mL dissolved in Tris-HCL buffer) were preincubated for 10 min at 37 °C, avoiding light exposure. Afterwards, 25 μL of MMP-2 substrate (50 μM dissolved in Tris-HCL buffer) were added, and the plate was incubated for an additional 30 min at 37 °C in the dark. The fluorescent intensity was measured at an excitation maximum of 320 nm and an emission maximum of 405 nm by Tecan (Infinite 200 Pro series). Crude extracts were evaluated at 75 μg/mL, 150 μg/mL, and 300 μg/mL, while pure compounds at 25 μM, 100 μM, and 500 μM were dissolved in PBS buffer, and the percentage of DMSO was under 5% of the total volume inside the plate. The negative control of the assay was 5% DMSO Tris-HCL buffer, and the positive control was Phosphoramidon (IC_50_ = 6.9 μM), a strong metallo-endopeptidase inhibitor. Experiments were performed in triplicate.

The collagenase inhibitory activity was calculated by the following equation:

Inhibition (%) = (F_A_ − F_B_)/(F_A_) × 100, (2)

where F_A_ = F_Control_ − F_Control Blank_ and F_B_ = F_Sample_ − F_Sample Blank_. F_Control_ is the fluorescence of Tris-HCL buffer with 5% DMSO, collagenase enzyme, and substrate, and F_Sample_ is the fluorescence of the tested sample in the Tris-HCL buffer, collagenase enzyme, and substrate. Blanks consist of all the above reagents except the enzyme.

#### 3.5.3. Statistical Analysis

GraphPad Prism software 8.4.3 was used for all the analyses. The means are represented as mean ± SD (*n* = 3) for all the biological assays, and the occurrence of the statistical differences among the data was evaluated by using one-way ANOVA. The IC_50_ calculation was performed using the four-parameter dose-response curve model.

## 4. Conclusions

This study presents a robust, eco-friendly, and scalable strategy for the efficient recovery and purification of the bioactive pentacyclic triterpenic acids, Oleanolic acid (OA) and Maslinic acid (MA), from olive leaves. The approach combines SFE for targeted extraction and fractionation with CPC for preparative-scale purification under pH-zone refining conditions. SFE, employed as the primary extraction tool, allowed the selective enrichment of OA and MA by modulating the polarity of supercritical CO_2_ with stepwise ethanol additions. To the best of our knowledge, this represents the first reported preparative-scale application of SFE for the concurrent extraction of both OA and MA from olive leaves, offering a new pathway for the utilization of this agricultural residue. Subsequent purification using pH-zone refining CPC enabled resolution of the two acids based on their acid-base properties, providing high purity and satisfactory yield of both compounds. Although a detailed cost-benefit analysis was beyond the scope of this study, it is worth noting that the combined use of SFE and CPC—despite higher initial equipment costs—offers significant long-term advantages, including reduced solvent consumption, shorter processing times, and higher product purity. These features support the scalability and industrial relevance of the method. Biological testing of the extracts and isolated compounds revealed notable inhibitory activity against elastase and collagenase. The OA- and MA-rich SFE fractions demonstrated effective elastase inhibition, with OA exhibiting a calculated IC_50_ of approximately 17 µM. In addition, this study provides the first reported activity of these triterpenes against MMP-2 collagenase, with all tested concentrations showing measurable inhibition. These results point to potential dermocosmetic applications and support further investigation into the therapeutic relevance of these compounds. The proposed workflow offers a viable approach to obtain bioactive compounds from olive leaves. The method is aligned with current efforts to valorize agro-industrial byproducts and provides a promising route for the development of functional ingredients in cosmetic and health-related sectors.

## Figures and Tables

**Table 1:** Yield of each extract at every time point.

	20 min	40 min	60 min
CO_2_	-	-	15.5 mg
CO_2_ + 2.5% EtOH	80.6 mg	54.4 mg	32.3 mg
CO_2_ + 5% EtOH	159.6 mg	118.8 mg	101.7 mg
CO_2_ + 7.5% EtOH	163.7 mg	109.5 mg	51.9 mg
CO_2_ + 10% EtOH	86.2 mg	79.4 mg	79.2 mg

## Data Availability

The raw data supporting the conclusions of this article will be made available by the authors on request.

## Supplementary Materials

The following supporting information can be downloaded at: https://www.mdpi.com/article/10.3390/molecules30132709/s1, Figure S1. HPTLC chromatograph of leaves extracts at every collecting time point. Figure S2. HPTLC chromatograms of olive leaf SFE extract analyzed using System A and System B, compared with pure OA and MA. Figure S3. HPLC-ELSD chromatograph of isolated pure MA. Figure S4. ^1^H-NMR spectrum of isolated pure MA. Figure S5. HPLC-ELSD chromatograph of isolated pure OA. Figure S6. ^1^H-NMR spectrum of isolated pure OA. Figure S7. HPLC-ELSD chromatograph of the starting raw material. Figure S8. Elastase inhibitory activity of isolated pure compounds (OA and MA). Bars represent mean ± S.D., *n* = 3., and Figure S9. Collagenase inhibitory activity of isolated pure compounds (OA and MA). Bars represent mean ± S.D., *n* = 3.
