# Supplementary material for: Rapid and Effective Recovery of Oleanolic and Maslinic Acids from Olive Leaves Using SFE and pH-Zone Centrifugal Partition Chromatography
Source: Molecules. 2025 Jun 24;30(13):2709. doi: 10.3390/molecules30132709 (PMC12250858; doi:10.3390/molecules30132709)
Supplement: Supplementary file 1 [file molecules-30-02709-s001.zip › molecules-3675140-supplementary.pdf]

Supplementary materials for the article

Rapid and Effective Recovery of Oleanolic and Maslinic Acids from Olive Leaves Using SFE and pH-Zone Centrifugal Partition Chromatography

Lemonia Antoniadi [1,2], Apostolis Angelis [2 *], Theodora Nikou [2], Dimitris Michailidis [1], Leandros A. Skaltsounis [2 *]

[1] PharmaGnose S.A., 57th km Athens-Lamia National Road, Oinofyta, 32011, Greece

[2] Division of Pharmacognosy and Natural Products Chemistry, Department of Pharmacy, National and Kapodistrian University of Athens, Panepistimioupoli Zografou, Athens, Greece

[*] Correspondence: aangjel@pharm.uoa.gr ; skaltsounis@pharm.uoa.gr

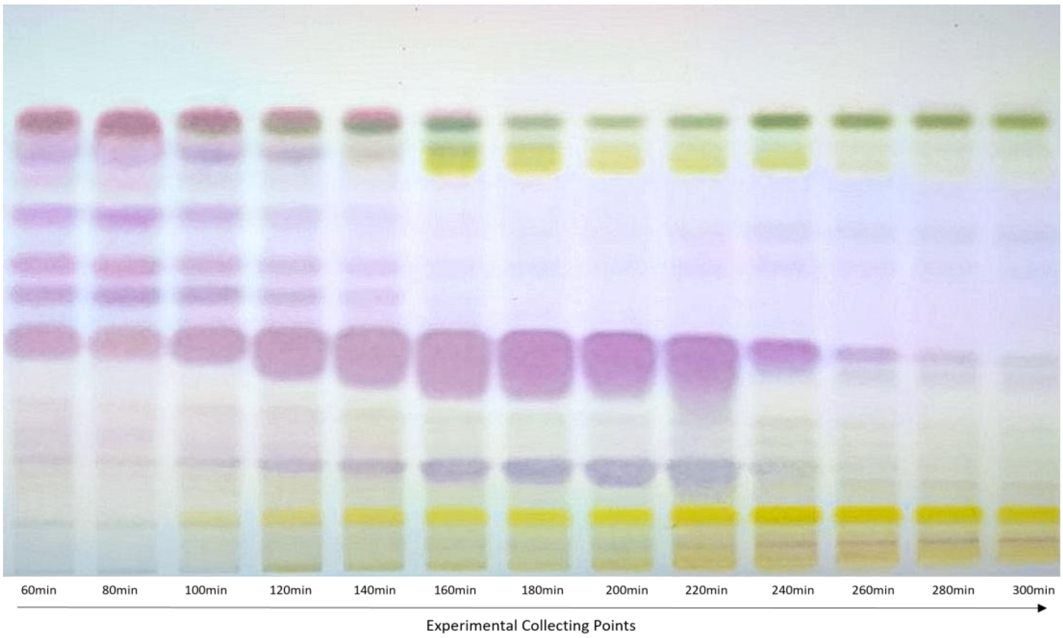

Figure S1. HPTLC chromatograph of leaves extracts at every collecting time point.

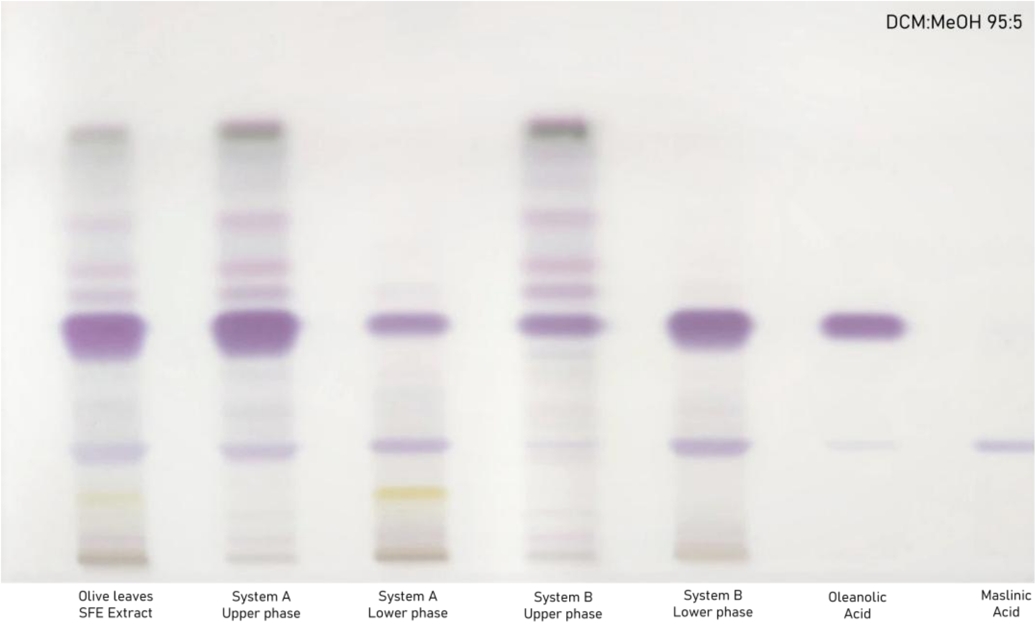

Figure S2. HPTLC chromatograms of olive leaf SFE extract analyzed using System A and System B, compared with pure OA and MA.

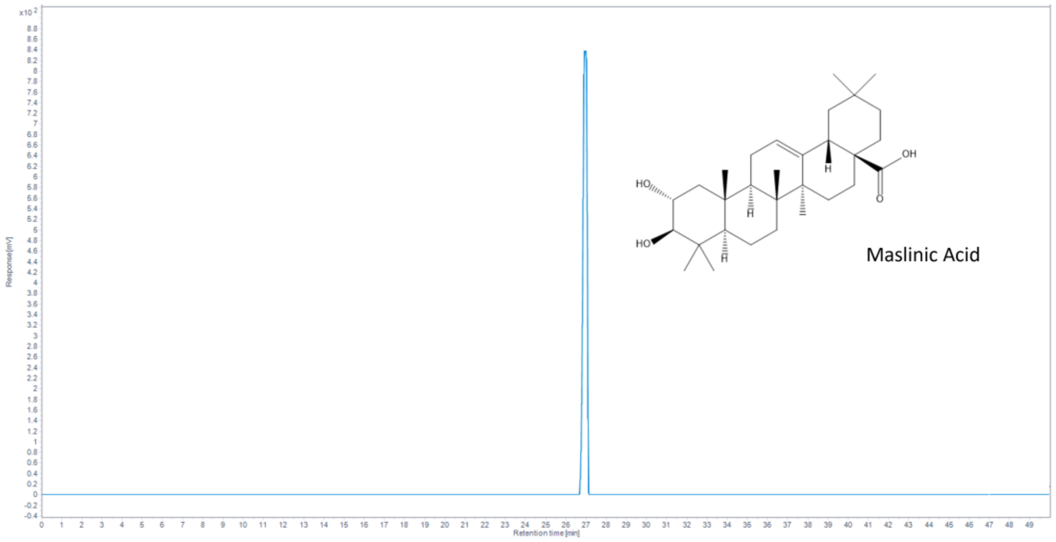

Figure S3. HPLC-ELSD chromatograph of isolated pure MA.

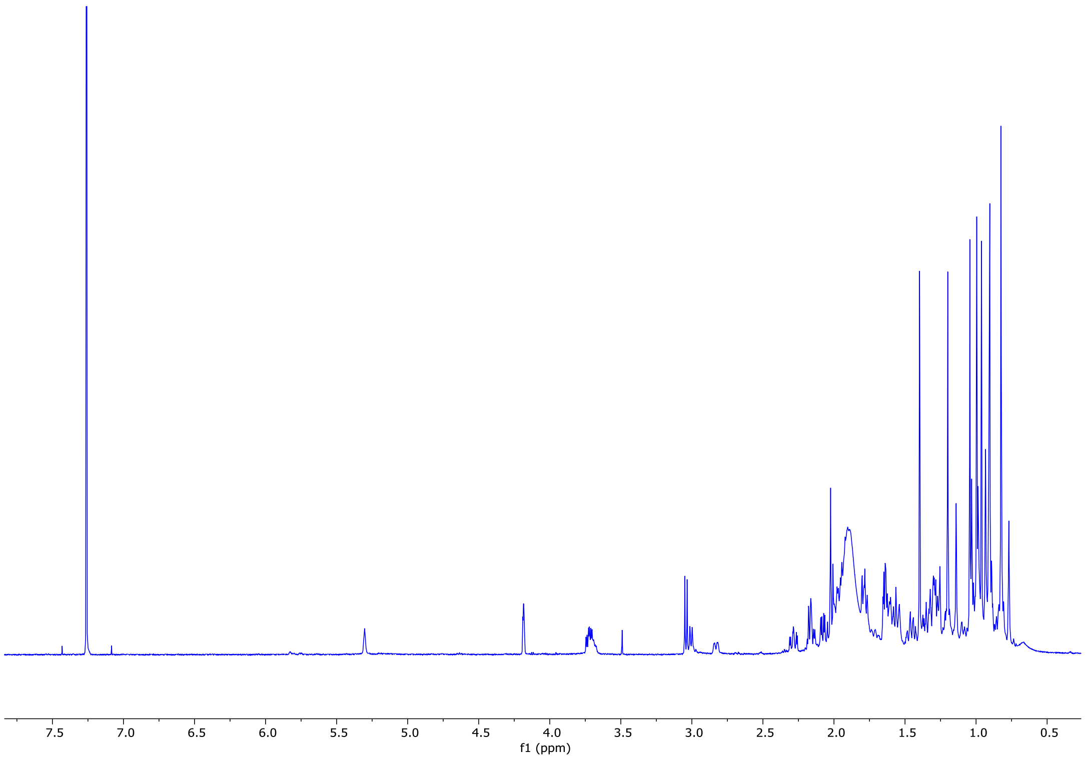

Figure S4. ^{1}H-NMR spectrum of isolated pure MA.

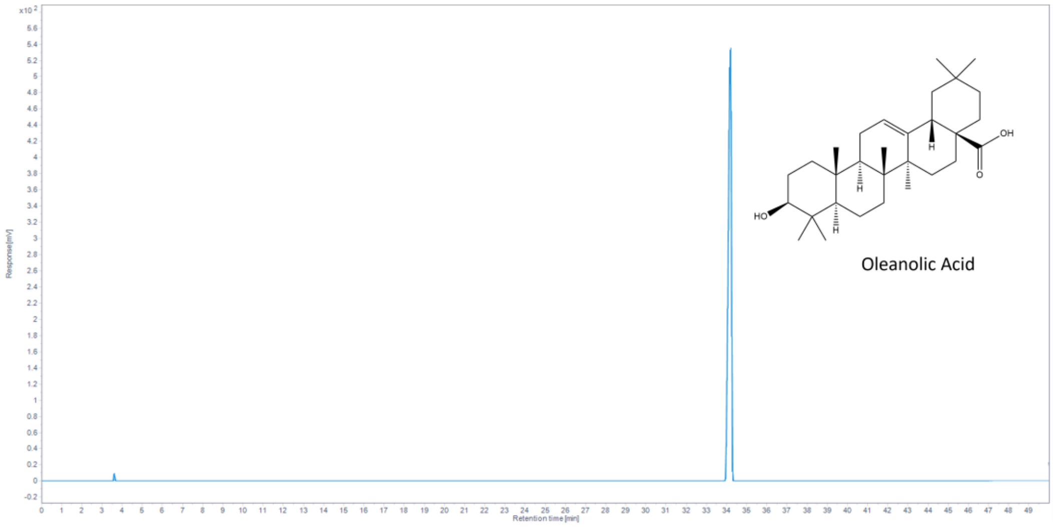

Figure S5. HPLC-ELSD chromatograph of isolated pure OA.

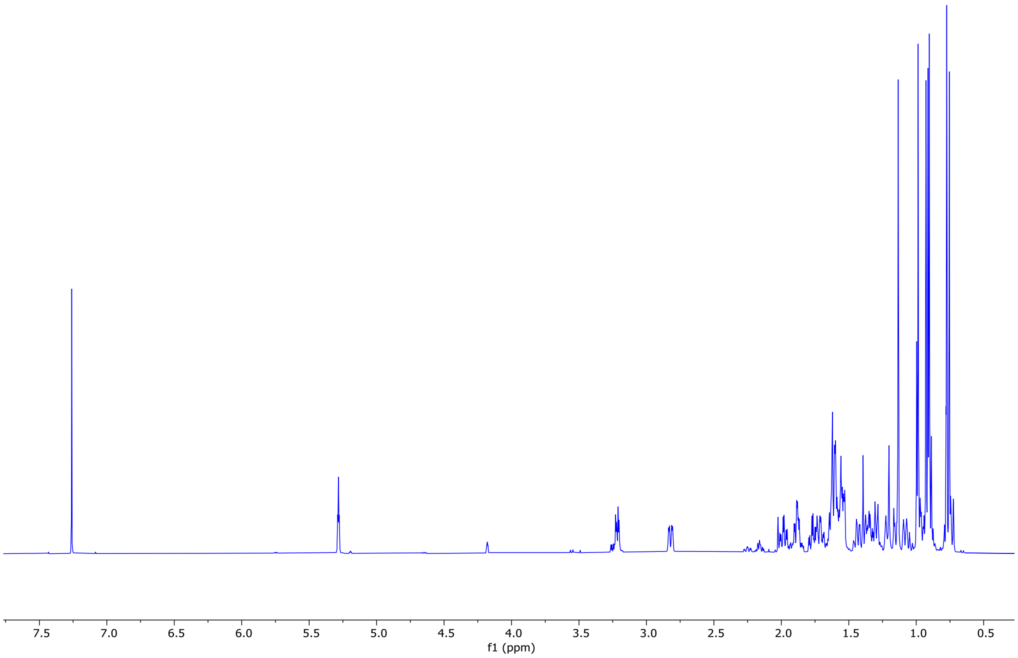

Figure S6. ^{1}H-NMR spectrum of isolated pure OA.

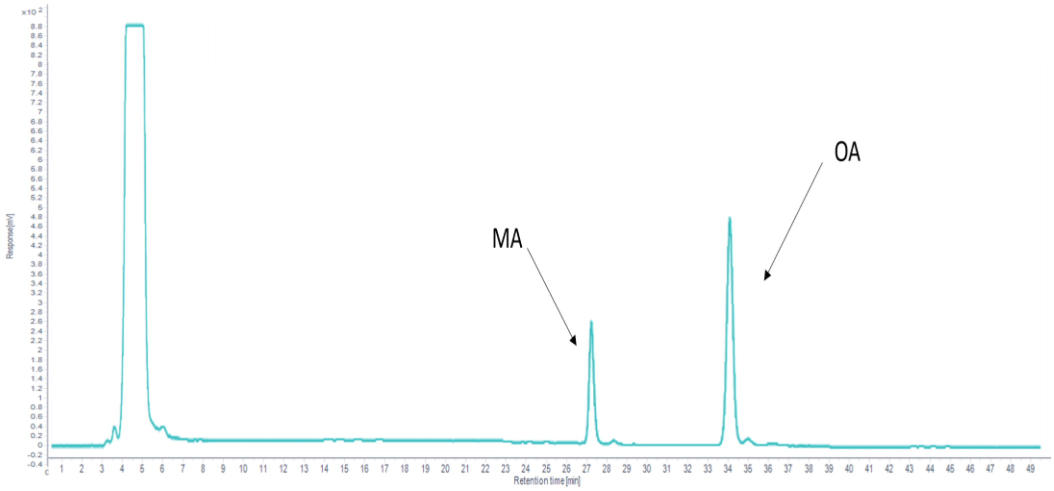

Figure S7. HPLC-ELSD chromatograph of the starting raw material.

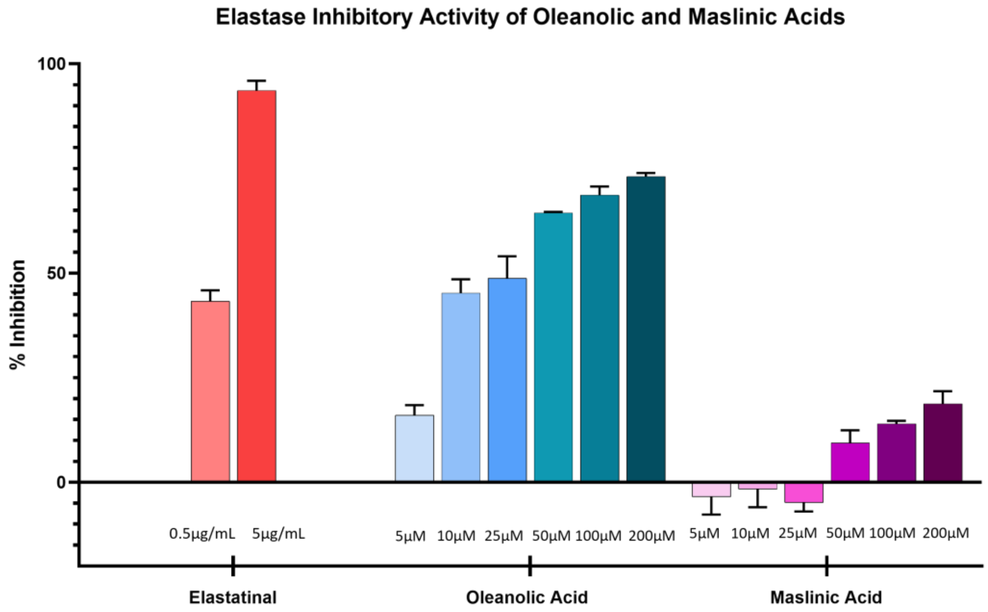

Figure S8. Elastase inhibitory activity of isolated pure compounds (OA and MA). Bars represent mean ± S.D., *n*=3.

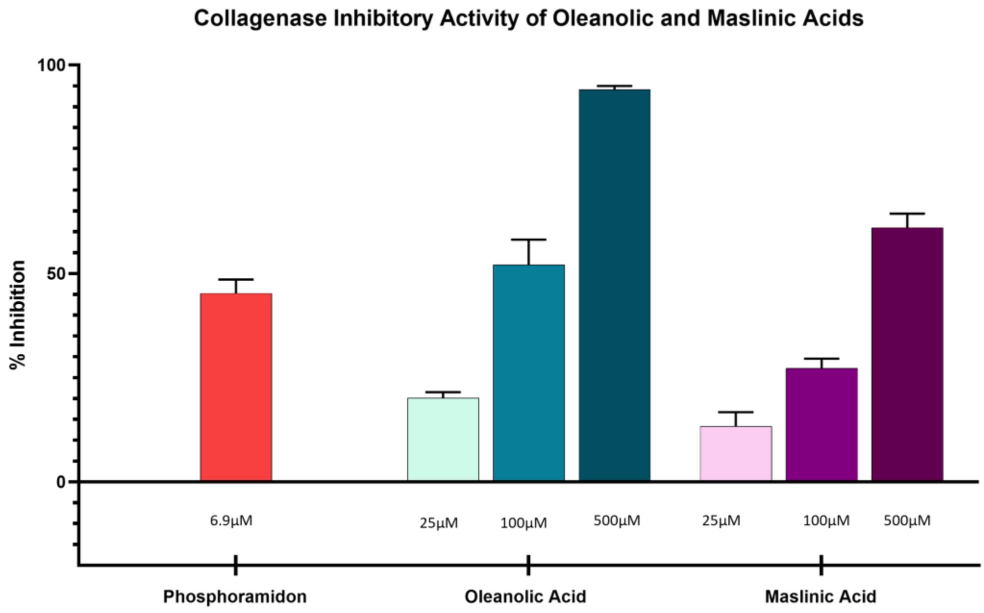

Figure S9. Collagenase inhibitory activity of isolated pure compounds (OA and MA). Bars represent mean ± S.D., *n*=3.
